# The effect of intra-articular botulinum toxin A on substance P, prostaglandin E_2_, and tumor necrosis factor alpha in the canine osteoarthritic joint

**DOI:** 10.1186/s12917-017-0990-y

**Published:** 2017-03-21

**Authors:** Helka M. Heikkilä, Anna K. Hielm-Björkman, John F. Innes, Outi M. Laitinen-Vapaavuori

**Affiliations:** 1 Department of Equine and Small Animal Medicine, Faculty of Veterinary Medicine, University of Helsinki, P.O. Box 57, Helsinki, 00014 Finland; 2 Department of Musculoskeletal Biology, Institute of Aging and Chronic Disease and School of Veterinary Science, University of Liverpool, Chester High Road, Neston, CH64 7TE UK; 3 Present address: ChesterGates Referral Hospital, CVS Vets Ltd., Units E and F Telford Court, Chestergates Road, Chester, CH1 6LT UK

**Keywords:** Intra-articular botulinum toxin A, Substance P, Prostaglandin E_2_, Tumor necrosis factor alpha, Osteoarthritis, Pain, Dog

## Abstract

**Background:**

Recently, intra-articular botulinum toxin A (IA BoNT A) has been shown to reduce joint pain in osteoarthritic dogs. Similar results have been reported in human patients with arthritis. However, the mechanism of the antinociceptive action of IA BoNT A is currently not known. The aim of this study was to explore this mechanism of action by investigating the effect of IA BoNT A on synovial fluid (SF) and serum substance P (SP), prostaglandin E_2_ (PGE_2_), and tumor necrosis factor alpha (TNF-α) in osteoarthritic dogs. Additionally, the aim was to compare SF SP and PGE_2_ between osteoarthritic and non-osteoarthritic joints, and investigate associations between SP, PGE_2_, osteoarthritic pain, and the signalment of dogs. Thirty-five dogs with chronic naturally occurring osteoarthritis and 13 non-osteoarthritic control dogs were included in the study. Osteoarthritic dogs received either IA BoNT A (*n* = 19) or IA placebo (*n* = 16). Serum and SF samples were collected and osteoarthritic pain was evaluated before (baseline) and 2 and 8 weeks after treatment. Osteoarthritic pain was assessed with force platform, Helsinki Chronic Pain Index, and joint palpation. Synovial fluid samples were obtained from control dogs after euthanasia. The change from baseline in SP and PGE_2_ concentration was compared between the IA BoNT A and placebo groups. The synovial fluid SP and PGE_2_ concentration was compared between osteoarthritic and control joints. Associations between SP, PGE_2_, osteoarthritic pain, and the signalment of dogs were evaluated.

**Results:**

There was no significant change from baseline in SP or PGE_2_ after IA BoNT A. Synovial fluid PGE_2_ was significantly higher in osteoarthritic compared to control joints. Synovial fluid PGE_2_ correlated with osteoarthritic pain. No associations were found between SP or PGE_2_ and the signalment of dogs. The concentration of TNF-α remained under the detection limit of the assay in all samples.

**Conclusions:**

The results suggest that the antinociceptive effect of IA BoNT A in the joint might not be related to the inhibition of SP nor PGE_2_. Synovial fluid PGE_2_, but not SP, could be a marker for chronic osteoarthritis and pain in dogs.

## Background

Osteoarthritis (OA) is the most common joint disease in dogs often cited to affect one fifth of the dog population over 1 year of age [1]. The most important clinical manifestation of OA is joint pain, which is characterized by hyperalgesia and pain at rest [2]. In a hyperalgesic joint the pain sensitivity is intensified because of sensitization in the nociceptive system. The sensitization is the result of a complex action of various inflammatory mediators, including pro-inflammatory cytokines, prostaglandins, and neuropeptides [2, 3].

Substance P (SP) is one of the principal neurotransmitters of pain in arthritis [2, 4, 5]. Substance P is produced in the cell bodies of the nociceptive afferent nerve fibers, and it is involved in the transmission of noxious stimuli from the joint into the spinal cord [4]. Following the activation of the afferent nerve fibers, SP is antidromically released from the nerves into the joint causing neurogenic inflammation and promoting peripheral sensitization [2, 4]. Increased level of SP in synovial fluid (SF) has been related both to OA and to joint pain in horses [6, 7], and the upregulation of SP-positive nerve fibers in the joint is associated with painful OA in human patients [8]. Nerve fibers containing SP have been found in various joint structures of dogs [9–11]; and recently, the concentration of SP in the spinal cord has been associated with central sensitization and pain in dogs with experimental OA [12]. However, to our knowledge the SF SP concentration and its association with osteoarthritic pain have not been studied in this species.

Prostaglandin E_2_ (PGE_2_) is a potent inflammatory mediator involved in both peripheral and central nociceptive pathways in OA [3, 13]. It is produced by the action of the cyclooxygenase (COX) enzymes in various cells [3]. The SF concentration of PGE_2_ correlates positively with osteoarthritic pain in dogs [14], horses [15], and humans [16, 17]. Substance P enhances the release of PGE_2_ from chondrocytes [18], and the concentrations of SP and PGE_2_ correlate with each other in the SF of osteoarthritic horses [6].

Tumor necrosis factor alpha (TNF-α) is one of the principal proinflammatory cytokines involved in the pathophysiology and pain of OA [3, 19, 20]. It is produced in various cells in the joint and it drives forward the inflammatory cascade by inducing the expression of other inflammatory and catabolic factors including PGE_2_ [20]. The SF TNF-α concentration is elevated in osteoarthritic dogs [21], horses [22], and humans [20]. The SF level of TNF-α correlates positively with pain in osteoarthritic human patients [23]. The correlation between the SF level of TNF-α and osteoarthritic joint pain has not been previously investigated in dogs.

Botulinum neurotoxin A (BoNT A) is a strong neurotoxin with antinociceptive effects. In the neuromuscular junction, BoNT A blocks the release of acetylcholine, which leads to muscle relaxation and pain relief [24]. However, the pain relief is not only related to the reduction in the muscle tone, but also to a direct antinociceptive effect of the toxin [24, 25]. In addition to acetylcholine, BoNT A inhibits the exocytosis of various other substances associated with inflammation and pain. Among others, BoNT A has been shown to inhibit the release of SP in rat dorsal root ganglia neurons [26] and in isolated rabbit iris muscles [27]; and BoNT A injections have suppressed pain, inflammation, and COX-2 expression in the prostate and bladder of rats [28, 29]. Finding the antinociceptive and anti-inflammatory effects of BoNT A has led to studies investigating its efficacy as an intra-articular (IA) injection in the treatment of arthritic pain in various species.

Intra-articular injection of BoNT A reduced joint pain in osteoarthritic dogs in our recent placebo-controlled, double-blinded clinical trial [30]. Similar results have been reported in human patients with arthritis [31–34], and in induced inflammatory arthritis in experimental horses and mice [31, 35]. Despite these promising results, the mechanism of the antinociceptive action of the toxin inside the joint has not been previously investigated.

The purpose of our study was to explore the mechanism of this antinociceptive action by investigating the effect of IA BoNT A on the concentrations of SP, PGE_2_, and TNF-α in the SF and serum of osteoarthritic dogs. The hypothesis was that their concentration decreases significantly after an IA BoNT A injection, which could explain the antinociceptive effect of the toxin in the joint. Additionally, our purpose was to compare the SF concentrations of SP and PGE_2_ between osteoarthritic and non-osteoarthritic control joints, and to investigate whether their concentration correlates with osteoarthritic pain in dogs. We also evaluated associations between these pain mediators and the signalment of the dogs.

## Methods

### Animals

Synovial fluid and serum samples were collected from privately owned osteoarthritic dogs. The dogs were included in our previous study on IA BoNT A in the treatment of osteoarthritic joint pain in dogs [30]. The study was approved by the Animal Experiment Board (ESAVI-2010-04178/Ym-23) and the Finnish Medicines Agency. The owners of the dogs signed an informed consent form having received information on the study. The inclusion criteria were chronic lameness present for at least 3 months, the diagnosis of OA in the stifle, elbow, or hip joint verified by radiographs, and pain on palpation of the joint. The exclusion criteria were lameness not related to OA, a neurological, systemic or infectious disease, age less than one year, and weight less than 15 kg. Also, the dogs were excluded if they had received any IA treatment, corticosteroids, or pentosan polysulphate injection in less than one month before the study, or treatment with nonsteroidal anti-inflammatory drugs or tetracyclines in less than 1 week before the study. The dogs were screened for participation in the study as described previously [30].

Synovial fluid samples were also collected from dogs without OA. These dogs served as non-osteoarthritic controls. The dogs were privately owned and donated for research at the University of Helsinki after euthanasia. The exclusion criteria were history of joint disease, age less than 1 year, weight less than 15 kg, and the above-mentioned medications. The sampled joints were confirmed healthy by SF analysis, macroscopical evaluation, and histopathological examination of biopsies taken from the weightbearing areas of the articular cartilage, the subchondral bone, and the synovium. The criteria for the histopathological assessment were modified from the OARSI Initiative [36]. The samples were excluded from the study, if there were any macroscopical alterations in the articular cartilage or synovium, if the total SF cell count was more than 2.0 × 10^9^/L [37], if there were more than 6% of neutrophils in the SF differential cell count [37], or if there were considerable abnormal findings in the histopathological examination.

### Study design

The study was carried out as a placebo-controlled, randomized, double-blinded clinical trial with stratified parallel group design. The dogs were stratified into six groups based on the administration of treatment into the stifle, elbow, or hip joint and on moderate or severe joint pain evaluated by Helsinki Chronic Pain Index (HCPI). The grouping for joint pain severity was based on the median of the HCPI results acquired from a screening visit. A moderate pain score was ≤16, and a severe pain score was ≥17. Randomization was performed using randomly permuted blocks. Dogs were randomized within each stratum in blocks of two in a 1:1 ratio to receive an IA injection of either 30 IU of BoNT A^1^ or an equivalent volume of placebo (0.3 mL sterile 0.9% saline) into the osteoarthritic joint. The randomization list was generated using SAS/Proc Plan^2^ and provided by a statistician to the research technician, who prepared the treatment for each dog following the list and covered the syringes with non-transparent tape. The dog owners, the veterinarians performing the trial, and the laboratory personnel participating in the analyses were masked to treatment allocation.

### Study procedure

#### Intra-articular injection and arthrocenteses

The osteoarthritic dogs were sedated for the IA injection. Sedation was accomplished by intramuscular injection of medetomidine (0.01 mg/kg) and butorphanol (0.1 mg/kg), or only butorphanol depending on the age of the animal. The sedation was followed by intravenous propofol anesthesia, if necessary. Synovial fluid and serum samples were obtained from the osteoarthritic dogs (IA BoNT A and placebo groups) before (baseline) and at 2 and 8 weeks after the IA medication.

The non-osteoarthritic control dogs were sedated by intramuscular injection of dexmedetomidine (10 μg/kg) and butorphanol (0.2 mg/kg) after which euthanasia was performed by intravenous propofol (10 mg/kg) and thiopental sodium (50 mg/kg). The SF sample was collected by arthrocentesis either from the stifle, elbow, or hip joint immediately after euthanasia. The joint was macroscopically evaluated after the arthrocentesis after which biopsies were taken and fixed in 10% neutral buffered formalin, embedded in paraffin wax, sectioned routinely, and stained with hematoxylin and eosin as well as toluidine blue for histopathological evaluation. The samples from bone were decalcified in EDTA-solution before embedding. No serum samples were collected from the control dogs.

### Processing of samples

The SF samples were processed within 30 minutes after the arthrocentesis. A part of the SF sample was separated into an EDTA tube for analyzing the total and differential cell counts. The rest of the sample was put into sterile Eppendorf-tubes, which were centrifuged at 10 000 rpm for 15 min. The supernatant was separated and stored in −80 °C.

The serum samples were taken into serum separating tubes and left to stand for 30 min, after which they were centrifuged at 3 500 rpm for 10 min. The serum was separated and stored in −80 °C.

### Sample analysis

The pain mediators were analyzed with commercially available ELISAs. The ELISAs for SP^3^ and PGE_2_ 4 are not validated for dogs, but because of the homologous nature of both molecules between species, the manufacturers report that these assays are suitable for evaluating canine samples. The ELISA for TNF-α^5^ is validated for canine serum and plasma, according to the manufacturer of the test.

All analyses were performed on both SF and serum. Samples were analyzed in duplicate according to the manufacturers’ instructions.

For SP and PGE_2,_ the assay accuracy was determined by evaluation of dilution parallelism. For TNF-α, the assay accuracy was determined by evaluation of a canine TNF-α control provided by the manufacturer and performing a spiking recovery test in SF and serum. Assay performance of each kit was monitored by the evaluation of a control SF sample included in each kit. All SF and serum samples of each dog were analyzed in the same plate of each assay.

### Substance P

The concentration of SP was measured with an assay^3^ based on a competitive binding technique, in which the intensity of the colorimetric signal is inversely proportional to the concentration of SP in the sample. The concentration of SP was reported as pg/mL. The detection limit of the assay was 3.9 pg/mL, as reported by the manufacturer.

For SP, the dilution parallelism was evaluated by assaying the sample undiluted and at dilutions 1:2, 1:5, and 1:10. The concentration of SP was higher in the diluted samples, which can be explained by an increase in the ratio of the high affinity antiserum to the SP binding proteins [38, 39]. Because our samples were on the low concentration range for this assay, the samples were assayed undiluted. No extraction was performed for the samples before the assay, because extraction results in loss of inconsistent amounts of SP [38, 39].

The intra assay coefficient of variation (CV) was 11.4% for SF and 11.8% for serum. The inter assay CV was 19.3%.

### Prostaglandin E_2_

The concentration of PGE_2_ was measured with an assay^4^ employing forward sequential competitive binding technique. In this technique, the intensity of the colorimetric signal is inversely proportional to the concentration of PGE_2_ in the sample. The concentration of PGE_2_ was reported as pg/mL. The detection limit of the assay was 13.4 pg/mL, as reported by the manufacturer.

For PGE_2_, the sample was assayed undiluted and at dilutions 1:2, 1:5, 1:10, and 1:20. Good assay linearity was achieved by dilutions. One in 20 was considered the optimal dilution for the SF samples and either 1:20 or 1:50 was considered the optimal dilution for the serum samples, depending on the concentration of PGE_2_. To test for a possible matrix interference in the PGE_2_ analysis, the linearity of the results from both diluted and non-diluted samples with and without extraction was evaluated. No extraction was considered necessary because of the consistent results of the extracted and unextracted samples. The samples were diluted in assay buffer.

The intra assay CV was 10.5% for SF and 9.1% for serum. The inter assay CV was 18.0%.

### Tumor necrosis factor alpha

The concentration of TNF-α was measured with an assay^5^ applying quantitative sandwich enzyme immunoassay technique, in which the intensity of the colorimetric signal is in proportion to the concentration of TNF-α in the sample. The concentration of TNF-α was reported as pg/mL. The detection limit of the assay was 2.4 pg/mL, as reported by the manufacturer.

A canine TNF-α control provided by the manufacturer was added in each plate to determine the assay accuracy. A spiking recovery test was performed by determining the recovery rate of this canine TNF-α control in SF and serum. Because the concentration of TNF-α remained below the detection limit of the assay in all the samples analyzed, no intra or inter assay CVs could be calculated.

### Clinical variables of osteoarthritic pain

The osteoarthritic pain was evaluated at baseline and at 2 and 8 weeks after the IA medication in the osteoarthritic dogs (IA BoNT A and placebo groups). The clinical variables of osteoarthritic pain were the ground reaction forces peak vertical force (PVF) and vertical impulse (VI), HCPI, and pain on palpation of the joint.

The ground reaction forces were measured with a force platform^6^and a computer software program^7^ at trot at a comfortable speed. Hind limb data were obtained from dogs with stifle and hip osteoarthritis, and fore limb data were obtained from dogs with elbow osteoarthritis. The acceptable range for the velocity of a trial was ± 0.5 m/s around the mean velocity of each dog and the acceptable range for acceleration was ≤ ±0.5 m/s^2^. The mean velocity of each dog was calculated when the study had ended and all trials had been obtained. Three to five valid trials were chosen for each dog at each visit, and their mean was used for analysis. All forces were normalized to bodyweight in kilograms. The HCPI questionnaire included questions regarding dog’s behaviour and demeanor during a 1 week period, as described by Hielm-Björkman [40], and it was always answered by the same dog owner. The veterinarian evaluated the pain on palpation of the joint using a five-point scale from 0 to 4 (0, no sign of pain; 1, mild pain (dog turns head in recognition); 2, moderate pain (dog pulls limb away); 3, severe pain (dog vocalizes or becomes aggressive); and 4, extreme pain (dog does not allow palpation).

The control dogs were not evaluated for osteoarthritic pain.

### Statistical analysis

All the continuously distributed variables were tested for normality using the Shapiro-Wilk test. Data were expressed as mean and standard deviation (SD) (normally distributed data) or as median and interquartile range (IQR) (non-normally distributed data). All of the statistical modelling was conducted using logarithmic transformed data for all of the pain mediators to normalize the distributions. The differences in the signalment of the groups of dogs (IA BoNT A, placebo, and controls) were analyzed using one-way analysis-of-variance (ANOVA) (continuous variables) or Fisher’s exact test (categorical variables). The differences between the treatment groups (IA BoNT A vs placebo) in change from baseline (at 2 and 8 weeks) in the mean SF and serum pain mediator concentrations were analyzed with linear mixed models for repeated measures (RM-ANCOVA). The models included the treatment group, time point, and the interaction of treatment group and time point as fixed terms, the baseline value of the pain mediator as a covariate, and dog as a random term. An unstructured covariance structure was applied in the model. Tukey-Kramer multiplicity adjustment method was used to correct the p-values of the multiple treatment comparisons. The differences in the SF pain mediator concentrations at baseline between osteoarthritic (pooled treatment groups) and control dogs were analyzed using Mann-Whitney U-tests. For the osteoarthritic dogs (pooled treatment groups) the differences in pain mediator concentrations between SF and serum at baseline were analyzed using Wilcoxon matched-pair signed rank tests. The correlations among the clinical variables of osteoarthritic pain and SF and serum pain mediator concentrations in osteoarthritic dogs (pooled treatment groups) were assessed by analyzing either the Pearson’s correlation coefficient (normally distributed data) or the Spearman’s rank correlation coefficient (non-normally distributed data) at baseline. The associations between SF and serum pain mediator concentrations and signalment of the dogs (IA BoNT A, placebo, and controls) were analyzed by using Fisher’s exact test. All tests were performed two-tailed and significance was set at *P* < 0.05. Statistical analysis was performed with statistical software^2^.

## Results

### Animals

Forty-eight dogs were included in the study. Thirty-five were osteoarthritic dogs, from which 19 were allocated to the IA BoNT A group and 16 to the placebo group. Thirteen were non-osteoarthritic control dogs. There were no significant differences between the groups of dogs in age, weight, gender, duration of lameness, or sampled joint (Table 1).

**Table 1:** Signalment of dogs

Variable	Osteoarthritic dogs		Control dogs
	IA BoNT A group	Placebo group	
	*n* = 19	*n* = 16	*n* = 13
Gender			
Female/neutered female	2/7	7/2	4/0
Male/neutered male	5/5	5/2	6/3
Weight (kg)			
Mean (SD)	33.0 (7.9)	33.2 (10.0)	34.9 (10.9)
Age (years)			
Mean (SD)	7.3 (3.0)	5.3 (3.1)	6.0 (3.2)
Duration of lameness			
3–< 6 months	1	2	Not applicable
6–12 months	2	6	
> 12 months	16	8	
Sampled joint			
Knee	6	5	7
Elbow	8	6	3
Hip	5	5	3
Breed (number of dogs)	Labrador retriever (6)	Labrador Retriever (4)	Mixed Breed (3)
	German Shepherd (3)	Bernese Mountain	German Shepherd
	Collie (2)	Dog (2)	(2)
	Basset Hound (1)	Nova Scotia Duck	Bernese Mountain
	Beauceron (1)	Tolling Retriever (2)	Dog (1)
	Belgian Shepherd (1)	Rottweiler (2)	Boxer (1)
	Cockerspaniel (1)	Black Russian	Bracco Italiano (1)
	Flat-Coated	Terrier (1)	Dalmatian (1)
	Retriever (1)	Catalan Sheepdog (1)	Dobermann (1)
	Irish Setter (1)	Central Asian	Great Dane (1)
	Rottweiler (1)	Shepherd Dog (1)	Rottweiler (1)
	Mixed Breed (1)	Siberian Husky (1)	Siberian Husky (1)
		Wales Springer	
		Spaniel (1)	
		Mixed Breed (1)	

### Substance P analysis

**Fig. 1:**
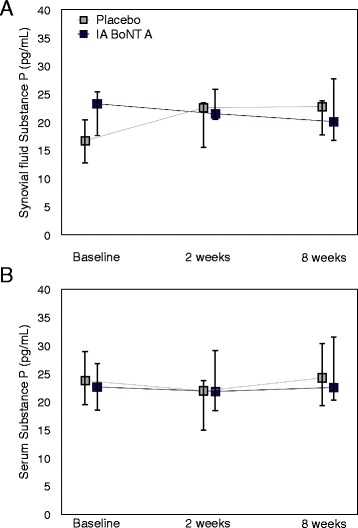
Synovial fluid (**a**) and serum (**b**) SP in osteoarthritic dogs treated with IA BoNT A or placebo. The concentrations are presented as median and interquartile range. A: *n* = 12 for the IA BoNT A group, *n* = 7 for the placebo group; B: *n* = 17, *n* = 16; respectively

In osteoarthritic dogs, SF and serum SP concentrations were not statistically different in the IA BoNT A group compared to the placebo group at baseline (*P* = 0.180 and *P* = 0.683, respectively). No significant change from baseline in SF or serum SP concentrations was found in either group during the study (*P* = 0.119 and *P* = 0.148 for overall change in SF and serum in the IA BoNT A group; *P* = 0.230 and *P* = 0.613 for overall change in SF and serum in the placebo group) (Fig. 1). No significant difference was detected between the IA BoNT A and placebo groups in the change from baseline during the study (*P* = 0.952 for SF and *P* = 0.176 for serum).

**Fig. 2:**
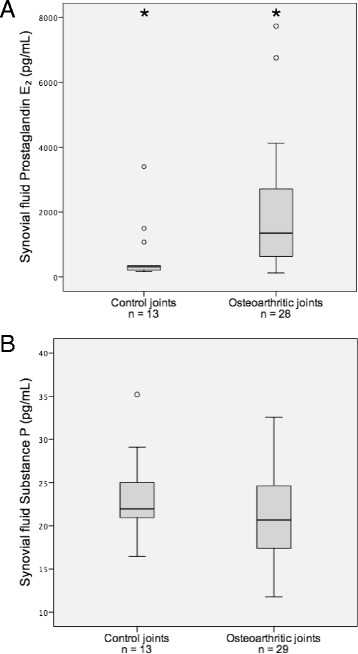
Synovial fluid PGE_2_ (**a**) and SP (**b**) concentrations in osteoarthritic and control joints. The central horizontal line indicates the median value and the boxes indicate the IQR. The top and bottom whiskers represent the highest and lowest case within 1.5 times of IQR, respectively. Values more than 1.5 times IQR are labelled outliers and represented as dots. *Values differ significantly (*P* = 0.001)

The SF SP concentration was not statistically different in osteoarthritic dogs compared to control dogs, *P* = 0.204 (Fig. 2).

### Prostaglandin E_2_ analysis

**Fig. 3:**
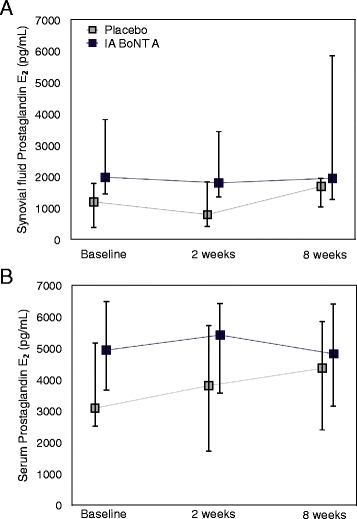
Synovial fluid (**a**) and serum (**b**) PGE_2_ in osteoarthritic dogs treated with IA BoNT A or placebo. The concentrations are presented as median and interquartile range. A: *n* = 12 for the IA BoNT A group, *n* = 7 for the placebo group; B: *n* = 17, *n* = 16; respectively

In osteoarthritic dogs, SF and serum PGE_2_ concentrations were not statistically different in the IA BoNT A group compared to the placebo group at baseline (*P* = 0.353 and *P* = 0.052, respectively). No significant change from baseline in SF or serum PGE_2_ concentrations was found in either group during the study (*P* = 0.105 and *P* = 0.907 for overall change in SF and serum in the IA BoNT A group; *P* = 0.726 and *P* = 0.863 for overall change in SF and serum in the placebo group) (Fig. 3). No significant difference was detected between the IA BoNT A and placebo groups in the change from baseline during the study (*P* = 0.475 for SF and *P* = 0.963 for serum).

The SF PGE_2_ concentration was significantly higher in osteoarthritic dogs compared to control dogs, *P* = 0.001 (Fig. 2).

### Tumor necrosis factor alpha analysis

In osteoarthritic dogs, both the SF and serum TNF-α concentrations were below the detection limit of the assay in all samples. Because of this, TNF-α was not measured from the control dogs.

### Correlations and associations

**Fig. 4:**
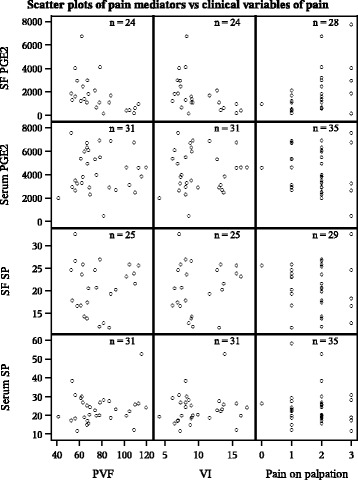
Scatter plots of synovial fluid and serum pain mediators vs clinical variables of pain in osteoarthritic dogs. Synovial fluid PGE_2_ was found to correlate with all the three clinical variables of pain, *P* < 0.05. No other correlations were detected. The correlations were measured in osteoarthritic dogs at baseline. SF = synovial fluid; PGE_2_ = prostaglandin E_2_; SP = substance P; PVF = peak vertical force, VI = vertical impulse

In osteoarthritic dogs, SF PGE_2_ concentration correlated negatively with the ground reaction forces PVF and VI and positively with pain on palpation of the joint at baseline (Fig. 4). Serum PGE_2_ correlated negatively with serum SP concentration. No other correlations were detected between serum pain mediators, SF pain mediators, and the clinical variables of osteoarthritic pain. The correlations are given in detail in Table 2. No associations were detected between the pain mediators and the age, weight, gender, duration of lameness, or the sampled joint.

**Table 2:** Spearman’s rank correlation coefficients between synovial fluid and serum pain mediators and clinical variables of osteoarthritic pain

Variable	SF PGE_2_	Serum PGE_2_	SF SP	Serum SP	PVF	VI	HCPI	Pain on palpation
SF PGE_2_	1.00	0.23	0.10	−0.05	−0.62*	−0.61*	0.05	0.45*
Serum PGE_2_	0.23	1.00	−0.11	−0.48*	0.03	0.04	−0.08	−0.14
SF SP	0.10	−0.11	1.00	0.09	−0.02	0.02	0.02	−0.09
Serum SP	−0.05	−0.48*	0.09	1.00	0.12	0.10	−0.03	−0.04

*SF* synovial fluid, *PGE*_*2*_ prostaglandin E_2_, *SP* substance P, *PVF* peak vertical force, *VI* vertical impulse, *HCPI* Helsinki Chronic Pain Index, Pain on palpation = subjective evaluation of pain on palpation of the joint. *P < 0.05

## Discussion

Intra-articular BoNT A reduces joint pain in osteoarthritic dogs [30] and human patients [31–33]. However, the antinociceptive mechanism of action of IA BoNT A in the joint is currently not known. In this study, we investigated the effect of IA BoNT A on SF and serum concentrations of SP, PGE_2_, and TNF-α in osteoarthritic dogs. We analyzed these pain mediators at baseline and at 2 and 8 weeks after treatment with IA BoNT A or placebo (0.9% saline).

The antinociceptive effect of IA BoNT A in the joint has been suggested to result from inhibition in the release of various inflammatory mediators and neurotransmitters [31, 32, 41]. However, this hypothesis is based on studies made in animal models and in vitro as no studies have been performed in a clinical setting neither in animals nor in humans. Botulinum toxin A has inhibited the release of SP, calcitonin gene-related peptide (CGRP), glutamate, and COX-2 in cell cultures [26, 42, 43], in tissue [27, 44–46], and in animal models of pain [28, 47, 48]. Our decision to measure SP was based on these findings. In addition, we measured PGE_2_ because of its association with osteoarthritic pain in dogs [14], and TNF-α due to its established role in the pathophysiology of osteoarthritic pain [2, 3, 20].

Somewhat surprisingly, we did not detect any significant change in the SF SP or PGE_2_ concentration in the IA BoNT A treated dogs during a follow-up time of 8 weeks. The explanation might be that contrary to the previous hypothesis, the antinociceptive effect of the toxin in the joint is not based on the inhibition of SP, nor in the inhibition of PGE_2_. Our hypothesis of the inhibition of PGE_2_ does not receive support from these data, although COX-2 is a key enzyme in PGE_2_ production [49] and BoNT A inhibits COX-2 [28, 29]. The toxin might have its effect by inhibiting other neurotransmitters, such as CGRP and glutamate, both of which are associated with pain in OA [8, 50].

Another explanation for not detecting a significant change in the pain mediator concentrations after IA BoNT A injection might be that the clinical effect of the toxin on pain was only mild. Although we have previously reported significant improvement from baseline in the IA BoNT A group compared to the placebo group after treatment [30], the pain relief might not have been strong enough to be detected as a significant change in the pain mediators’ concentrations.

In addition to the effects of IA BoNT A on SF and serum pain mediators, we also compared the SF PGE_2_ and SP concentrations between healthy joints and joints with chronic, naturally occurring OA in dogs. We also investigated whether their concentration correlates with clinical variables of osteoarthritic pain and whether any associations exist between the pain mediators and the age, weight, gender, duration of lameness, or the sampled joint.

The PGE_2_ concentration was significantly higher in osteoarthritic compared to healthy dogs (*P* = 0.001). Previously, elevated PGE_2_ concentration has been reported in osteoarthritic joints of horses [6, 51] and an increase in SF PGE_2_ has been documented in experimental dogs after cranial cruciate ligament transection [14]. It has been speculated, that the level of SF PGE_2_ would peak in the early phase of OA [6, 14]. However, our results show, that the SF PGE_2_ concentration is also significantly increased in chronic OA in dogs.

We detected a positive correlation between the SF PGE_2_ concentration and joint pain in osteoarthritic dogs at baseline. The SF PGE_2_ correlated negatively with the ground reaction forces PVF and VI (r = −0.619, *P* = 0.001, and *r* = −0.613, *P* = 0.001; respectively), which indicates less weight bearing on joints with higher concentration of PGE_2_. In addition, the SF PGE_2_ concentration correlated positively with pain on palpation of the osteoarthritic joint (*r* = 0.446, *P* = 0.017). This is in accordance with the study by Trumble et al. [14], in which the SF PGE_2_ concentration correlated with various pain measures in dogs with experimental OA, and with the study by van Loon et al. [15], in which it correlated with pain and lameness in horses with experimentally induced synovitis. Positive correlation between PGE_2_ and an index of pain, stiffness, and physical disability has also been reported in osteoarthritic human patients [16]. To the authors’ knowledge, this correlation has not been previously studied in dogs with chronic, naturally occurring OA.

Contrary to the SF PGE_2_, serum PGE_2_ did not correlate with the clinical variables of pain in our study. Serum PGE_2_ was approximately threefold higher than the SF PGE_2_ (*P* = 0.000) in the osteoarthritic dogs, which suggests that the local production of PGE_2_ is important for the perception of joint pain in OA in dogs and that the serum PGE_2_ level is affected by other factors. Therefore, our study suggests that SF but not serum PGE_2_ might have a role as a marker of chronic pain in naturally occurring OA in dogs.

In contrast, SF SP appeared not to be a good indicator for osteoarthritic joint pain in our study. Contrary to the studies in horses [6, 7] and humans [8], we did not detect any significant difference in the SF SP between osteoarthritic and healthy joints. Also, we found no correlation between the degree of joint pain and the SF SP in osteoarthritic dogs. Our finding is in accordance with the study by van Loon et al. [15], in which IA morphine reduced lameness without affecting the SF SP concentration in horses. However, contradictory results have also been reported [7]. A positive correlation between SP and joint pain has been documented in human patients [52], but no correlation [53, 54], and a negative correlation has also been published [55]. In dogs, the concentration of SP in the cerebrospinal fluid has been associated with pain in syringomyelia [56], but to the authors’ knowledge, its concentration in SF and its association with joint pain has not been previously studied in this species.

We found a negative correlation between the serum PGE_2_ and SP concentrations (*r* = −0.478, *P* = 0.004). This was unexpected, and might not be of clinical relevance. Previously, a positive correlation has been reported between SP and PGE_2_ in SF in horses [6].

We did not detect any TNF-α in the SF or serum samples of the osteoarthritic dogs in our study, and because of this, we did not measure TNF-α from the non-osteoarthritic control dogs. However, to rule out a problem in the assay, we determined the recovery rate of a known amount of TNF-α in the SF and serum samples and were able to obtain consistent results. Also, in a study by Carter et al. [57] TNF-α activity was detected in only 2 out of 80 osteoarthritic canine SF samples. Using a variety of assay methods, previous studies have found higher [21] and lower [58] concentrations of TNF-α in osteoarthritic compared to normal joints of dogs; and in the light of these findings, it would have been interesting to measure TNF-α also from the control dogs in our study. The discrepancy between the results of the previous studies has been explained by loss of TNF-α activity during storage, presence of specific inhibitors in the SF, transient peaks in TNF-α activity during the disease, and different methods of sample preparation [21, 57, 58]. However, not detecting any TNF-α in our osteoarthritic dogs might also be explained by the chronicity of the disease, as the level of TNF-α has previously been reported to be higher in acute severe joint disease compared to joint disease in general [59], but conflicting findings have also been recently published [22, 60].

The number of samples in our pain mediator analyses in the IA BoNT A and placebo groups was quite small (*n* = 12 and *n* = 7, respectively), because we were not able to collect enough SF for the analyses from all dogs at every visit. We collected raw SF samples and opted not to use a lavage method [61] to increase sample volume because we did not want the lavage to interfere with the biomarker analysis or with the effects of the toxin.

One limitation in our study is that although the healthy joints in our study went through a very thorough evaluation to exclude any disease process, we did not check the contralateral joints in all of these animals. Four out of the 13 healthy joint samples were pooled samples from both stifle, elbow, or hip joints. This was necessary to provide sufficient SF for the analysis, because the amount of SF was very small in some healthy joints. In the non-pooled samples, there is a possibility that a disease process in the contralateral joint would have affected the concentration of the SF pain mediators in the joint used as a healthy control in our study. This is especially noteworthy for SP, because the levels of this neurotransmitter have been reported to interrelate with each other in bilateral joints via a neurogenic mechanism [62, 63].

A further limitation in our study is that in the process of preparing this manuscript, the manufacturer of the SP ELISA reported finding a 100% cross-reactivity between SP and a recently found neuropeptide hemokinin-1 (HK-1) in human samples in their assay. Hemokinin-1 binds to the same receptors as SP with similar affinity [64] and shares biological activities common to SP [65]. However unlike SP, it is primarily expressed in non-neuronal tissues [65, 66]. The biological relevance of HK-1 is still unclear, but some of the physiological activity previously assigned to SP might in fact be that of HK-1, especially in the non-neuronal tissues [65, 67]. The cross-reactivity was reported in human samples, but it is not known whether it applies to dogs. To date HK-1 and other novel SP-like peptides are described in man, rat, and rabbit [66], but to the best of our knowledge, not yet in dogs.

## Conclusions

Contrary to the previous hypothesis, our results suggest that the antinociceptive effect of IA BoNT A in the joint might not be related to the inhibition of the release of SP or to the release of PGE_2_. In addition, our findings indicate that SF PGE_2,_ but not serum PGE_2_, could be a marker for chronic OA and pain in dogs. However, neither SF nor serum SP seem to be good markers of osteoarthritic pain in this species.

## Abbreviations

BoNT A: Botulinum neurotoxin A
CGRP: calcitonin gene-related peptide
COX: Cyclooxygenase
HCPI: Helsinki Chronic Pain Index
HK-1: Hemokinin-1
IA: Intra-articular
IQR: Interquartile range
OA: Osteoarthritis
PGE_2_: Prostaglandin E_2_
PVF: Peak vertical force
SD: standard deviation
SF: Synovial fluid
SP: Substance P
TNF-α: Tumor necrosis factor alpha
VI: Vertical impulse

## Footnotes

1 Botox, Allergan, Inc., Irvine, CA

2 SAS for Windows, versions 9.2 and 9.3, SAS Institute, Inc., Cary, NC

3 Substance P EIA Kit, Chayman Chemical Company, Ann Arbor, MN

4 PGE_2_ ELISA Kit, Enzo Life Sciences, Inc., Farmingdale, NY

5 Quantikine ELISA, Canine TNF-α, R&D Systems, Inc., Minneapolis, MN

6 Kistler force plate, type 9286, Kistler Instrumente AG, Amherst, NY

7 Acquire 7.3, Sharon Software Inc., Dewitt, MI
